# Urban sprawl and microclimate in the Ga East municipality of Ghana

**DOI:** 10.1016/j.heliyon.2022.e09791

**Published:** 2022-07-02

**Authors:** Kwasi Frimpong, Darko Eugene Atiemo, E.J. Van Etten

**Affiliations:** a School of Governance and Public Services Ghana Institute of Management and Public Administration, Accra, Ghana; b Centre for Ecosystem Management, School of Science, Edith Cowan University, Perth, WA, Australia; c School of Medical and Health Sciences, Edith Cowan University, Perth, WA, Australia

**Keywords:** Microclimate, Rainfall, Temperature, Urban sprawl, Ga east municipality

## Abstract

Climatic elements such as temperature and rainfall provide great and unquantifiable benefits to human health. However, rapid urban sprawl has the tendency to undermine these health consequences. The relationship between urban sprawl and microclimate in the Ga East Municipality has been assessed to present the extent of sprawl that inhibit temperature and rainfall in recent times. Methodologically, satellite imagery and meteorological data (minimum and maximum temperature and rainfall) from 1990 to 2020 were used. The results indicate that rapid urban sprawl in recent times has significantly undermined the local climate through land use and land cover changes. There was strong statistical relationships between temperature and built-up areas (p < 0.05), grass/shrub cover (p < 0.04) and all vegetation cover (p < 0.03). There was also strong statistical relationship between rainfall and built-up areas (p < 0.03), grass/shrub cover (p < 0.04) and all vegetation (p < 0.02). Thus, expansion in built up areas and reduced grass/shrub cover led to increases in temperature, rainfall and surface water run off while reduction in all vegetation led to increase in both temperature and rainfall. These changes in climate brought about by urban sprawl will affect crop production, increase cataclysmic floods as well as growth of some harmful insects. There is the need for the amalgamation of urban growth and climate change into spatial planning through an all-embracing approach.

## 1 Introduction

Human health continues to receive unquantifiable benefits from climatic elements such as rainfall, temperature, relative humidity among others. However, these elements are highly responsive to anthropogenic activities such as rapid urbanization and urban sprawl (Mensah, 2020; Li et al., 2017). Whereas urbanization refers to the transition of the social order from rural to urban resulting from migration, urban sprawl is the expansion of the geographic space in a form of built up, extensive road networks and other paved surfaces (Mansouri Daneshvar, 2019; Manu et al., 2006) ensuing from urbanization and population growth (Emadodin et al., 2016; Bekele, 2005). The impact of these activities leads to numerous climatic consequences (Patra et al., 2018; Li et al., 2017), with developing countries becoming more vulnerable to these changes (Fentahun and Gashaw, 2014).

The linkage between urban sprawl and climate change/variability has gained global attention in recent decades due to high rise in urban population (Saghir and Santoro, 2018; Feng and Gauthier, 2021; Ewing and Hamidi, 2015), high occurrence rates of cataclysmic floods, torrential rainfall, extreme heat waves and exposure (Frimpong et al., 2017; Nunfam et al., 2021), elevated wildlife extinction, rising sea or ocean levels and increases in energy consumption (Waseem et al., 2021; IPCC, 2014; Battista and de Lieto Vollaro, 2017). Rainfall and temperature variations are seen to be connected to changes in LULC (Fu and Weng, 2016; Cao et al., 2015). For instance, replacing vegetation with asphalt and concrete in urban surfaces has the potential to accumulate abundant amount of heat (Patra et al., 2018), lower evapotranspiration (Feng and Gauthier, 2021, European Environment Agency, 2016), increase surface runoff (Paul et al.*,* 2018; Zhang et al. 2017), and reduce moisture content thereby reducing precipitation (Emadodin et al., 2016; Adler and Coauthors, 2017).

Over the last three decades in Ghana as exemplified in recent studies (Frimpong et al., 2017; Nunfam et al., 2021), climate change has been characterized by intensifying temperatures, diminishing but unpredictable rainfall, mounting sea levels and rampant extreme weather and disaster (World Bank Data, 2017; MEST, 2010). With an average yearly increase of 1 °C temperature over the last three decades, it is projected that average yearly temperature will rise between 0.8 °C and 5.4 °C for the years 2020 and 2050 while rainfall will decrease by between 1.1% and 20.5% within the same years for all agro-ecological zones (Owusu and Waylen 2013). These changes in climate are largely human induced (Yao et al., 2015; Rosenzweig, 2007; Sachs, 2008); for instance, over the few years urban sprawl has resulted in conversion of vegetation and grassland into built-up areas (Langemeyer et al., 2021; Afriyie et al., 2019; Berthal et al., 2019; Abass et al., 2018) and increased fuel consumption from both electricity production and transport (Feng and Gauthier, 2021), with the overall effect being increased carbon dioxide emissions (Asante and Amuakwa-Mensah, 2015; Saboori et al., 2014). In studying the relationship between sprawl and climate change, the use of Geo-spatial techniques are relevant as they give precise and accurate changes in the various land uses (Waseem et al., 2021), as well as lessening climatic risks and trepidations in the fast urban development process (Wang et al.*,* 2015*)*. While a lot of researches have been conducted on urban sprawl effects and climate change worldwide (Liu and Niyogi, 2019; Mansouri Daneshvar et al.2019; Alqurashi and Kumar, 2017; Chapman et al., 2017; Deng et al., 2013), very few studies have been conducted in Ghana with the past research foci being on the extent of sprawl, green space reduction and policy implementation to reduce urban sprawl (Cobbinah et al., 2019; Daniels et al., 2018; Mensah, 2020; Mattah et al., 2018), livelihood and ecological management (Afriyie et al., 2019), environmental sustainability (Yiran, 2020), and loss of agricultural lands (Abass et al., 2018; Cobbinah and Aboagye, 2017). Amazingly, no preceding work has scrutinized the impact of urban sprawl on microclimate in the Greater Accra region and its Ga East Municipality in particular. This article therefore is assessing the impact of urban sprawl on microclimate using climatic variables of rainfall and temperature. The study also sought to identify the trend of urban sprawl, rainfall and temperature variability in Ga East Municipality and to examine the extent to which urban sprawl has impacted microclimate.

## 2 Study location and methodology

**Figure 1:**
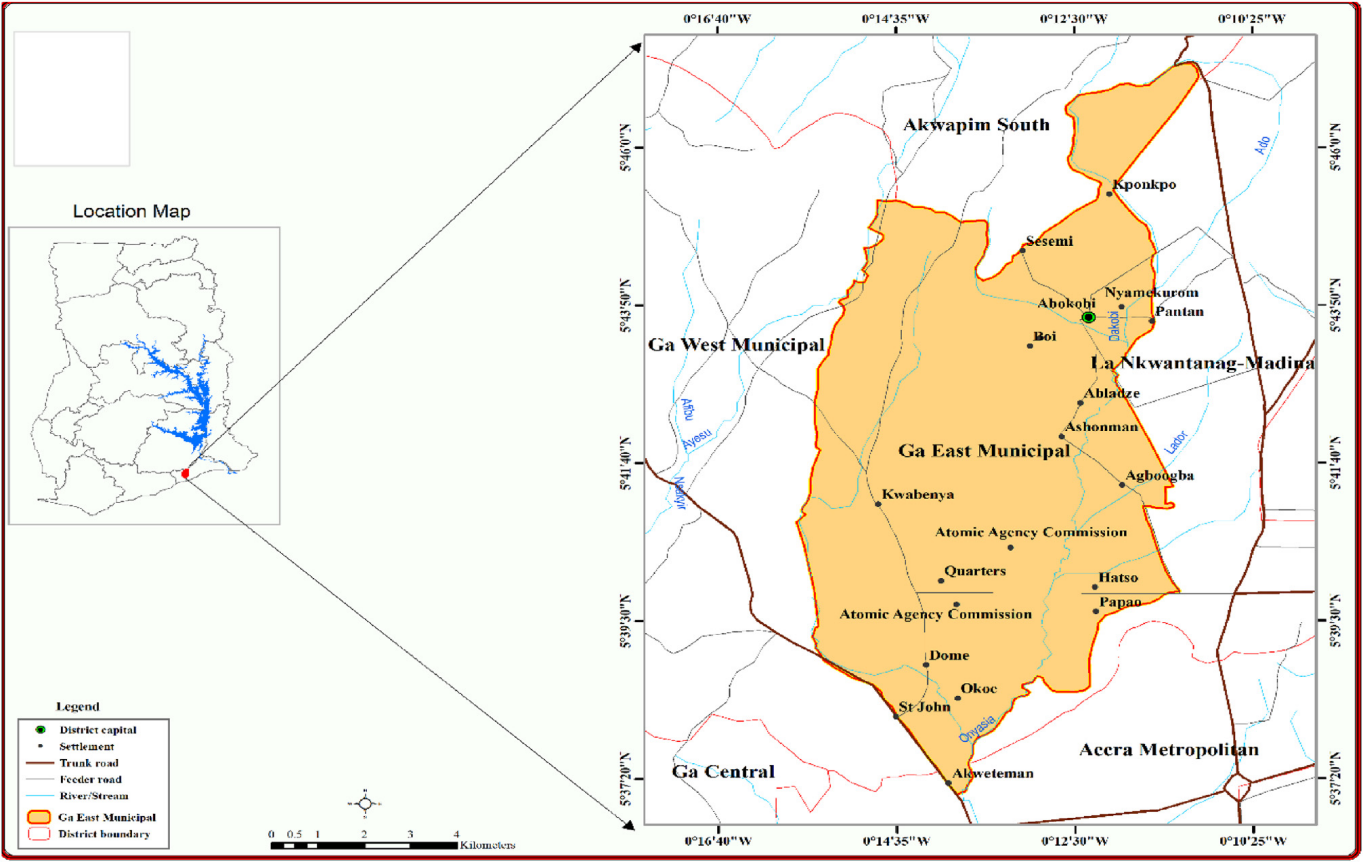
Map of the study area.

The Ga East Municipality is among the sixteen (16) Municipalities of the Greater Accra Region situated at the north between latitudes 5°36' and 5°47'N, and Longitude 0°15' and 0°11'W. In terms of area, it stretches over an area of 96 km^2^ and it is surrounded by the Ga West Municipality on the west, La—Nkwantanang—Madina Municipality on the east, while on the north and south, the Municipality share borders with Akuapem South District and the Ayawaso West Municipality respectively. The capital of the Municipality is Abokobi and the entire area is sub divided into two administrative zonal councils, namely the Abokobi and Dome Zonal Councils (Ga East Municipal Assembly, 2018). The municipality lies in the savannah agro-ecological zone with shrub and grassland vegetation. The topography consists of gentle slopes, but the Akuapem range rises steeply above the western end and lies between 37m and 420 m north of Aburi and falls to 300m southward. Rainfall is bimodal with major rainy season between April and June and the minor season spanning from September to November. The average annual temperature ranges between 25.1 °C in August and 28.4 °C in February to March. March is seen as the hottest month while August is observed as the coldest month (Ga East Municipal Assembly, 2018; Asante and Amuakwa-Mensah, 2015). The population of the area is projected to reach 186,342 by 2020 and it is 87.5% urbanized. Migration inflow is the main cause of population growth in the municipality. The population structure comprises about 51% males and 49% females with an average household size of 4.6. The four main economic activities in the Municipality are agriculture, industry, service and commerce (GSS, 2018; Ga East Municipal Assembly, 2018) (see Figure 1).

### 2.1 Remote sensing data application and analysis

Landsat satellite images for the years 1990, 2000, 2010 and 2020 obtained from United States Geology Survey (USGS) were utilized. They were used because of their conveniency in storing long-term images with average spatial resolution and dependable spectral and radiometric resolutions. Summary information about the dataset used is listed in Table 1. Acquisition of the images was confined to those captured during cloud-free weather.

**Table 1:** Summary information of the datasets used in the analysis.

Satellite	Sensor	WRS Path/Row	Spectral Resolution	Spatial Resolution	Date Acquisition	Source
Landsat 7	ETM	193/056	7 bands	30 x 30	10th Jan. 1990	Earthexplorer.usgs.gov
Landsat 7	ETM	193/056	7 bands	31 x 30	5th Mar. 2000	Earthexplorer.usgs.gov
Landsat 7	ETM	193/056	7 bands	32 x 30	30th Jan. 2010	Earthexplorer.usgs.gov
Landsat 8	OTI_TIRS	193/056	11 bands	33 x 30	14th Feb. 2020	Earthexplorer.usgs.gov

The bands of the spatial images were stacked for easy conversion from panchromatic to multispectral images, with the help of Erdas Imagine 2014 image processing software. The images were classified by sorting their pixels into finite number of individual classes. Pixels that satisfied their set of criteria were assigned to the class corresponding to the criteria. The supervised classification method which ensures greater accuracy (Desa, 2014) was adopted to facilitate the generation of spectral signature using maximum Likelihood Algorithm with the knowledge of the Area of Interest (AoI) in mind (Abass et al., 2018). New class value numbers were assigned in a form of recoding to all classes of the same spectral characteristics leading to the creation of new thematic raster layer. These attributes were later exported to excel to generate the statistics of the area. Finally, times series maps were composed using ArcMap software to exhibit the changes in the cover types of 1990, 2000, 2010 and 2020. Five cover types were identified: the closed thicket herbaceous, the open thicket herbaceous, the dense herbaceous, the grass/shrubs vegetation and the bare surface/built up areas. Change detection analysis was employed to categorize and compute the alterations between the classified time series images (Wiafe and Asamoah, 2018). The 1990 Classified images were initially matched with 2000 while 2000 was also matched with 2010. Then 2010 classified images were compared with 2020. The annual rate was computed by subtracting the area of land cover for the initial year (*AreaiYearx)*from the area of land cover for the subsequent year of the following data (*AreaiYearx+1)* and dividing the result by the number of years between the initial year and subsequent years (*tYears*) (Mensah, 2020; Kaishaigili and Majaliwa, 2010). Accuracy assessment was also carried out on the classified image using error matrix to describe how near the categorization agreed with the image that were checked directly in the field (Othow et al., 2017; Mosammam et al., 2017) and also serve as a pathfinder for quality and consistent map (Mensah, 2020). Sample points to be validated during the field visitation was carried out, taking into consideration their uniqueness. In all (150) reference points on the classified image were generated by ArcMap software but 120 points were validated during the filed visitation: Closed thicket herbaceous (25), Open thicket herbaceous (30), dense herbaceous (25), Grass/shrubs (25) and Bare/built up areas (15). The Germin Etrex GPS was used in collecting point source data in geographic coordinates when sites were visited and were further exported to excel for the overall accuracy, user’s and producer’s errors to be determined. The Overall accuracy is the whole accuracy of the classified images and it is usually expressed as a percent, with 100% accuracy being a perfect classification where all reference site were classified correctly. User’s accuracy basically informs the user how often the class on the map will truly be present on the ground. Producer accuracy similarly portrays how real features on the ground are correctly shown on the classified map or the probability that a certain land cover of an area on the ground is classified as such. Error of commission is the prospect of an explicit class which is incorrectly classified on the ground. Whereas Error of omission is the prospect of an explicit class to be incorrectly classified in the reference data. The formulae for calculating these accuracies are stated below

(1) $$\text{Annual}\;\text{rate}\;\text{of}\;\text{change}=\frac{\text{AreaiYearx}+1-\text{AreaiYearx}}{\text{tYears}}$$

(2) $$\text{Over}\;\text{all}\;\text{accuracy ​}=\frac{\text{Total}\;\text{number}\;\text{of}\;\text{correctly}\;\text{classified}\;\text{sites}\;}{\text{Total}\;\text{number}\;\text{of}\;\text{reference}\;\text{sites}}\times \;100$$

(3) $$\text{User's}\;\text{accuracy ​}\;\;\;\;=\;\;\text{​}\frac{\text{Correctly}\;\text{classified}\;\text{reference}\;\text{sites}\;}{\text{Total}\;\text{number}\;\text{of}\;\text{classified}\;\text{sites}}\times \;100$$

(4) $$\text{Producer's}\;\text{accuracy}\;\;=\;\;\frac{\text{Correctly}\;\text{classifies}\;\text{reference}\;\text{sites}\;}{\text{Total}\;\text{number}\;\text{of}\;\text{reference}\;\text{sites}}\times \;100$$

(5) $$\text{Error}\;\text{of}\;\text{omission}\;\;\;=\;\;\frac{\text{Incorrectly}\;\text{classified}\;\text{reference}\;\text{sites}\;}{\text{Total}\;\text{number}\;\text{of}\;\text{reference}\;\text{sites}}\times \;100$$

(6) $$\text{Error}\;\text{of}\;\text{commission}\;=\;\;\frac{\text{Incorrectly}\;\text{classified}\;\text{reference}\;\text{sites}\;}{\text{Total}\;\text{number}\;\text{of}\;\text{classified}\;\text{sites}}\times \;100$$

### 2.2 Meteorological data

Climatic data made up of temperatures (minimum and maximum) and rainfall were acquired from Ghana Meteorological Agency (GMet) covering thirty years (1990–2020). These data were grouped into three decadal years: 1990–2000, 2000–2010 and 2010–2020. Daily maximum and minimum temperatures were averaged to get the mean monthly maximum and minimum temperatures. By averaging the monthly maximum and minimum temperatures, the annual mean temperatures were calculated. With regards to rainfall, the annual rainfall was calculated by summing up monthly rainfall. Graphs were used to depicts the changing trends over the years while statistical analysis was conducted to measure any significant difference that has taken place among these variables. The mean temperature was regressed against the matching rainfall data. Correlation analysis was performed to identify the association between urban sprawl and the climatic elements.

The Mann-Kendall (MK) test was also employed to establish the presence of statistically significant trend in the minimum and maximum temperature and the annual average rainfall figures (Mavromatis et al., 2011; Wang et al., 2015; Drapela and Drapelova, 2011).

### 2.3 Normalized Difference vegetation index (NDVI)

Normalized Difference Vegetation Index (NDVI) is the most common vegetation index used globally to represent vegetation health and coverage (Lee et al., 2021; Taddeo et al., 2021; Grover and Singh 2015; Huang et al., 2020). In Landsat satellite image, Near-Infrared (NIR) band (band 4) and the Red bands were used for the NDVI calculation. NDVI was calculated using the formula below:

(7) $$\text{NDVI}=\frac{R_{NIR}-R_{red}}{R_{NIR}+R_{red}}$$

Where $R_{NIR}$ is the near infrared reflectance band and $R_{red}$ is the red spectral reflectance band. The resultant values after NDVI calculation ranges from +1 to −1 (Rhyma et al., 2020; Huang et al., 2020 Yengoh et al., 2016). The positive NDVI value suggest a healthy green vegetated area, whereas the negative or lower NDVI values signify a low or non-vegetated area.

### 2.4 Land Surface Temperature (LST)

Land Surface Temperature (LST) was also employed and used the thermal band in the Landsat images (Wang et al., 2020). The digital numbers (DN) into radiance values, the radiance was converted into temperature brightness (Degree Kelvin), and finally converting the temperature brightness into LST (Degree Celsius). The algorithms used are shown in Eqs. (8) and (9) below:

(8) $$L_{\lambda }=\frac{Lmax_{\lambda }-Lmin_{\lambda }}{\left(Qcal_{max}-Qcal_{min}\right)}*\left(Qcal-Qcal_{min}\right)+\;Lmin_{\lambda }$$

(9) $$T=\frac{K_{2}}{ln\left(\frac{K_{1}}{L_{\lambda }}+1\right)}$$

Where: T = temperature in Kelvin *K*, $K_{2}$= calibration constant in degrees Kelvin, $K_{1}$ = The calibration constant in Watts/(m^2^ ∗ sr ∗ μm) and $L_{\text{λ}}$= The spectral radiance from equation.

The result from Eq. (9) was converted into Degree Celsius using Eq. (10)

(10) C=T−273.15

## 3 Results

### 3.1 Accuracy assessment using matrix errors

Eqs. (2), (3), (4), (5), and (6) assisted in finding out the accuracy assessment, thus comparing the classified results with ground truth data. The overall accuracy for 2020 was 82.5% signifying high agreement with the ground reference data. From the assessment, the producer’s accuracies are 84.0%, 83.3%, 84.0% 80.0% and 80.0% while user’s accuracies 91.3%, 83.3%, 75.0%, 83.3% and 80.0% for closed thicket herbaceous, open thicket herbaceous, dense herbaceous, grass/shrub and bare/built-up areas respectively. Within the same period the computed errors of omission were 16.0%, 16.7%, 16.00%, 20.0% and 20.0% and that of error of commission were 8.7%, 16.7%, 25.0%, 16.7% and 20.0% for the classes of closed thicket herbaceous, open thicket herbaceous, dense herbaceous, grass/shrub and bare/built-up areas respectively. The elucidation for these statistics is that closed thicket herbaceous and dense herbaceous verified the highest producer accuracies (84.0%) followed by open thicket herbaceous (83.3%) whiles grass/shrubs and built-up areas recorded the least (80.0%). This specifies that the “Closed thicket and Dense herbaceous” classes had the highest probability of being correctly classified. In similar way, for the user’s accuracy, closed thicket herbaceous had the highest (91.3%) was followed by open thicket herbaceous and grass/shrub (83.3%), then built-up areas (80,0%) and dense herbaceous (75.0%). The indication is that closed thicket herbaceous had the maximum likelihood of what was classified on the map truly represents exactly what is on ground.

### 3.2 Land use and land cover change analysis

**Figure 2:**
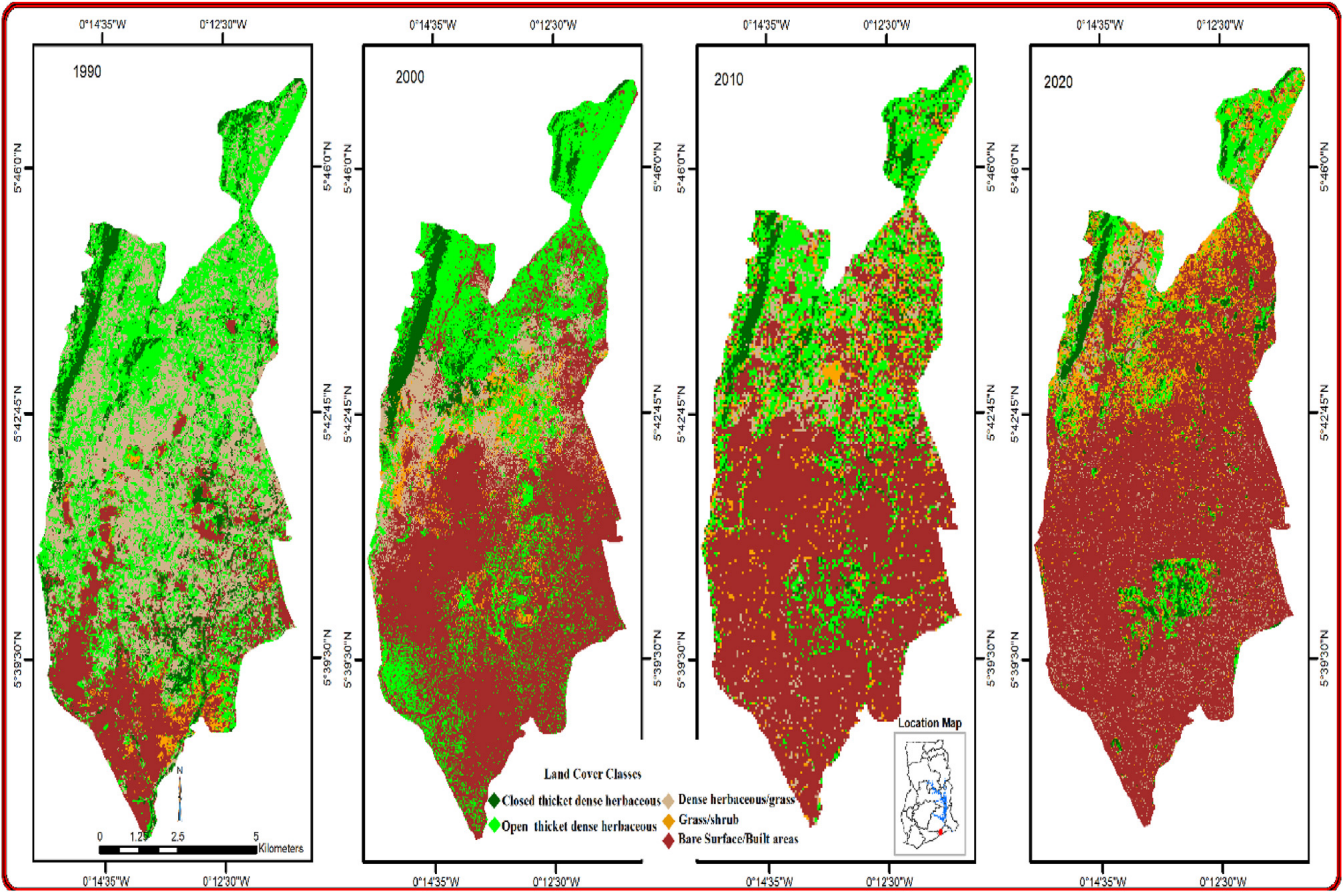
Land cover pattern –urban sprawl.

As displayed in Figure 2 and Table 3, 1990, 2000, 2010, and 2020 images of the study area were classified. It can be observed that the area under different LULC classes have changed over the years. With regard to the land cover classes, for the past three decades closed thicket, open thicket and dense herbaceous have decreased respectively by 5.21 km^2^, 24.27 km^2^ and 23.92 km^2^ at average rates of −5.21 km^2^, −0.81 km^2^ and −0.79 km^2^. Whereas grass/shrub and built-up areas have increased by 8.0 km^2^ and 45.44 km^2^ at average rates of 0.27 km^2^ and 1.52 km^2^ respectively within the same period. Regression analysis was also done to trajectory the trends of the changes in each individual LULC class. It came to light that the three LULC classes (closed thicket, open thicket and dense herbaceous) have noted declining trend with very high R^2^ values of the trend equations (0.6371), (0.994), and (0.716) respectively. On the other hand, grass/shrubs and built-up areas showed increasing trend with R^2^ values of 0.989 and 0.876 respectively.

**Table 2:** Constant values of Eq. (10).

Constant	Landsat 4TM	Landsat 5TM	Landsat 7 ETM+
K1	671.62	607.76	666.09
K2	1284.30	1260.56	1282,71

**Table 3:** Land change analysis.

Land cover type	Area (Sq. km)				Annual rate of change (Sq. km/year)			
	Sl	2000	2010	2020	1990–2000	2000–2010	2010–2020	1990–2020
Closed thicket herbaceous	9.43 (11%)	4.46 (5%)	4.27 (5%)	4.22 (5%)	−0.50	−0.02	−0.01	−0.17
Open thicket herbaceous	32.44 (36%)	25.69 (29%)	18.38 (21%)	8.17 (9%)	−0.68	−0.73	−1.02	−0.81
Dense herbaceous	28.99 (32%)	8.47 (9%)	6.49 (7%)	5.07 (6%)	−2.10	−0.19	−0.14	−0.79
Grass/shrubs	1.62 (2.0%)	3.86 (4%)	7.48 (8%)	9.62 (11%)	0.22	0.36	0.21	0.27
Bare/built up areas	16.94 (19%)	46.93 (52%)	52.8 (59%)	62.34 (70)	2.99	0.59	0.95	1.51

Figure 4 Map shows the high and low vegetation NDVI for the Ga East Municipality over a thirty-year period (1990–2020). In 1990, 0.380 was recorded as the high vegetation index while −0.013 was recorded as the lowest vegetation index. In 2000, 0.232 and 0.0472 were recorded as the high and low vegetation indexes respectively. By 2010 the high vegetation index was 0.077 and the low index was −0.325. while 2020 had 0.457 and 0.0147 being the high and low vegetation index. The highest vegetation index recorded from 1990 to 2020 is 0.457 and was recorded in 2020. However, 1990 recorded the least vegetation index. A negative NDVI value indicates the likelihood of water. The “Odaw” river takes it source from Abokobi and Adjankote hills through Ashongman and the Atomic energy (all in the Ga East Municipality) before entering other municipalities. A value close to +1 tells of the possibility of dense green leaves while NDVI with the value close to Zero demonstrates the possibility of urbanized area. From Figure 4 even though most of the values are positive, they are approaching zero than +1, hence urbanized. Thus urban built up has taken a greater part of the municipality.

### 3.3 Land Surface Temperature

The use of satellite technique with thermal sensors has afforded easy observation and measurement of Land Surface Temperature which hitherto was difficult to observe with the real eyes (Walawender et al., 2014). The application of the technique has unraveled the spatio-temporal variations in temperature (LST) at different decades within the Ga East Municipality and the results are presented. The results indicate that both maximum and minimum LST increased initially, fell and rose again within the study area. In 1990, the maximum and minimum temperatures of 26.96 °C and 21.56 °C were obtained. In 2000, 33.16 °C and 22.27 °C were also observed as maximum and minimum temperatures. In 2010, 30.53 °C and 21.4 °C were recorded as the maximum and minimum temperature. In 2020, maximum temperatures gave a value of 31.97 °C while minimum temperature was 21.01 °C. During 1990–2020, the highest temperature changes were observed between 1990 and 2000 (6.2 °C) then between 2010 and 2020 (1.45 °C) and finally the least temperature occurred between 2000 and 2010 (−2.73 °C). The highest increase in the mean LST (3.46 °C) occurred between 1990 and 2000 while the least increase in mean LST (−1.76 °C) occurred between 2000 and 2010. This is attributed to rapid increase in built up areas (16.94–46.93 sq.km) within the same year (Table 3). Since LST of a study area depends meaningfully on the LULC types, areas with green vegetation have low LST value while the built-up areas and bare lands have moderate to high LST value.

### 3.4 Decadal variations in temperature and rainfall

**Figure 3:**
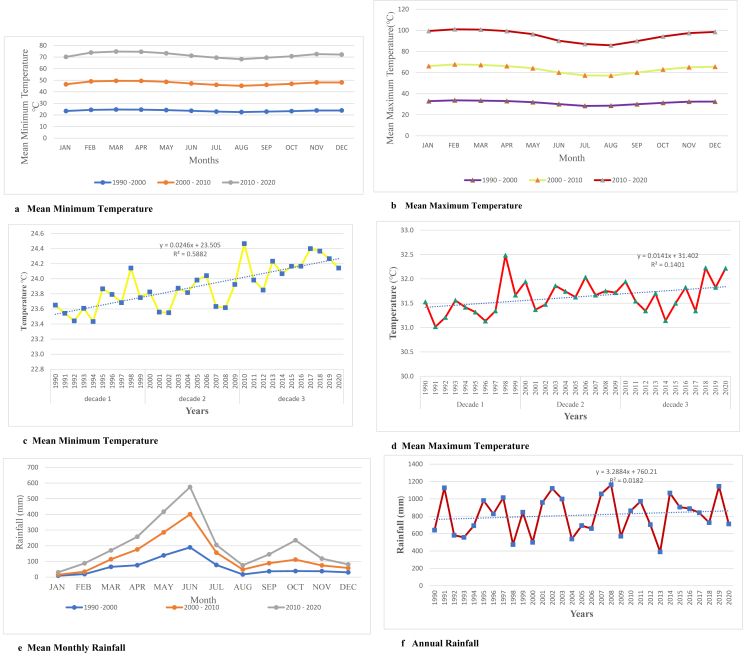
a. Mean minimum temperature; b. Mean maximum temperature; c. Mean minimum temperature; d. Mean maximum temperature; e. Mean monthly rainfall; f. Annual rainfall.

Figure 3a, b, c, d, e, f and Table 3 graphically demonstrate temperature and rainfall patterns from 1990 to 2020. During the first era (1990–2000), mean monthly minimum and maximum temperatures recorded 23.7 °C and 31.5 °C respectively, these figures rose significantly in the second era (2000–2010) to 23.9 °C and 31.7 °C respectively. For the third era (2010–2020), mean monthly maximum temperature maintained a constant increase of 31.7 °C while mean monthly minimum temperature rose to 24.2 °C. In effect 2010–2020 experienced the warmest period since both mean monthly minimum and maximum temperatures were high. For all the three decadal years, the highest maximum temperatures were recorded in February (33.6°) and March (33.7 °C) and the least maximum temperatures occurring in July (29.0 °C) and August (28.6 °C). In terms of minimum temperature, the highest mean months were February (24.6 °C), March (24.9 °C), and April (24.5 °C) while the least minimum temperature was recorded in August (22.7 °C) within the same period. With regards to rainfall (Table 3 and Figure 3e), the second decadal period (2000–2010) received the highest average amount of rainfall (69.0 mm) followed by the third decadal period (69.7 mm) and then the first decadal period (60.9 mm). In other words, from 1990 all the way through 2020, the average rainfall amounts increased by 8.8 mm with the highest change in average (8.0 mm) recorded within 2000–2010 decadal years. The major rainy months were April with an average rainfall of 85.8 mm, May (139.1 mm) and June (191.5 mm), while the minor rainy months were September (48.4 mm), October (78.2 mm) and November (39.1 mm). This pattern together with the amount received changed gradually, for instance in third decades more rains were received in the minor seasons than the first and second decades’ minor seasons. The Municipality is typified by very low rainfall during the months of January, February and December and this coincides with high temperature recordings.

### 3.5 Relationship between temperature and rainfall

A regression analysis to institute the connection between rising temperature and rainfall disclosed that when temperature increased, rainfall amount also increased. The high R^2^ value of 0.9947 (significant at the p < 0.002) indicates that intensifying temperatures could lead to higher rainfall amounts and variations in rainfall pattern with unpredictable effects including reduction food security as a result of low agriculture productivity, loss of natural habitat for organisms and higher incidence rate floods among others.

### 3.6 Mann- kendall test trend

Figures 3c, d and f are the graphs for mean minimum and maximum temperature observations for Ga East Municipality. On running the Mann-Kendall test on temperature and rainfall data, the null hypothesis (H_0_) of “no trend present” is tested against the alternative hypothesis (H_0_) of “trend present.” The results from the test show that there exists a significant trend in the Minimum Temperature (p < 0.001) whilst the behavior of Maximum Temperature (p < 0.281) and Rainfall (p < 0.068) recorded over the period shows no significant trend (see Table 4).

**Table 4:** Mean minimum, maximum monthly temperatures and rainfall for Ga East Municipality.

Month	1990–2000			2000–2010			2010–2020		
	TPN	TPX	R’FALL	TPN	TPX	R’FALL	TPN	TPX	R’FALL
JAN	23.4	32.9	8.4	23.1	33.2	6.3	23.7	33.3	15.7
FEB	24.4	33.7	19.0	24.6	34.0	15.1	24.9	33.3	53.4
MAR	24.7	33.4	65.1	24.8	33.8	48.4	25.3	33.5	56.9
APR	24.6	33.0	75.4	24.8	33.1	100.7	25.2	33.2	81.3
MAY	24.2	31.9	138.0	24.4	32.2	147.1	24.7	32.3	132.2
JUN	23.6	30.1	189.3	23.6	29.9	211.2	24.0	30.1	174.0
JUL	22.9	28.4	76.9	23.1	28.9	78.8	23.5	29.7	49.0
AUG	22.5	28.6	16.7	22.7	28.5	31.2	23.0	28.7	27.1
SEP	22.9	30.0	36.5	23.1	30.0	51.8	23.5	29.8	56.8
OCT	23.3	31.3	38.1	23.6	31.6	73.1	23.8	31.3	123.5
NOV	23.9	32.4	37.3	24.2	32.6	36.8	24.5	32.4	43.3
DEC	23.9	32.5	29.8	24.2	33.0	27.9	24.1	32.9	23.0

TPN = Minimum Temperature, TPX = Maximum Temperature, R’FALL = Rainfall.

## 4 Discussion

### 4.1 Land use change analysis

To fully appreciate the degree of cover changes over the 30 years, the annual rate of change was used (Eq. (1)). Change detection exploration demonstrates the variations that occur at an area between a time frame (Mensah, 2020; Kaishaigili and Majaliwa, 2010). Commencing from 1990 to 2000 (Table 3) paramount areas were transformed to built-up areas by 2.99 km^2^/year and was accompanied grass/shrubs 0.22 km^2^/year while dense herbaceous, open and closed thicket were lost by −2.10 km^2^/year, −0.49, and −0.68 respectively. The greatest cover loss was the dense herbaceous. Between 2000 and 2010 an interesting LULC change was observed. Though Grass/shrub and built-up maintained their dominance land covers (0.36 km^2^/year and 0.59 km^2^/year), built up increased at a diminishing rate while grass/shrub increased an increasing rate. Open thicket (−0.73 km^2^/year) overtook dense herbaceous (−0.19 km^2^/year) in terms of cover loss with closed thicket reducing marginally. Throughout the 30 years, built up and grass/shrubs increased at the expense of other land cover types. This corroborates Bhat et al. (2017) that speedy growths in built-up in Dehradun city (India) leads to the modifications of land use and cover as exemplified by rapid decrease in agriculture, fallow and vacant areas. The causes of these land cover loss can be attributed principally to migration and population increase as a result of urbanization from the adjoining districts. The municipality is among the areas within the region with availability of lands hence procuring lands has now become competitive. This result is similar to earlier studies by Ashiagbor et al. (2019) and Biney and Boakye (2021) in which they all informed that the continuous growth in the built-up areas of most developing cities like Accra and Takoradi over the last thirty years were connected to the rapid urbanization and population growth.

**Figure 4:**
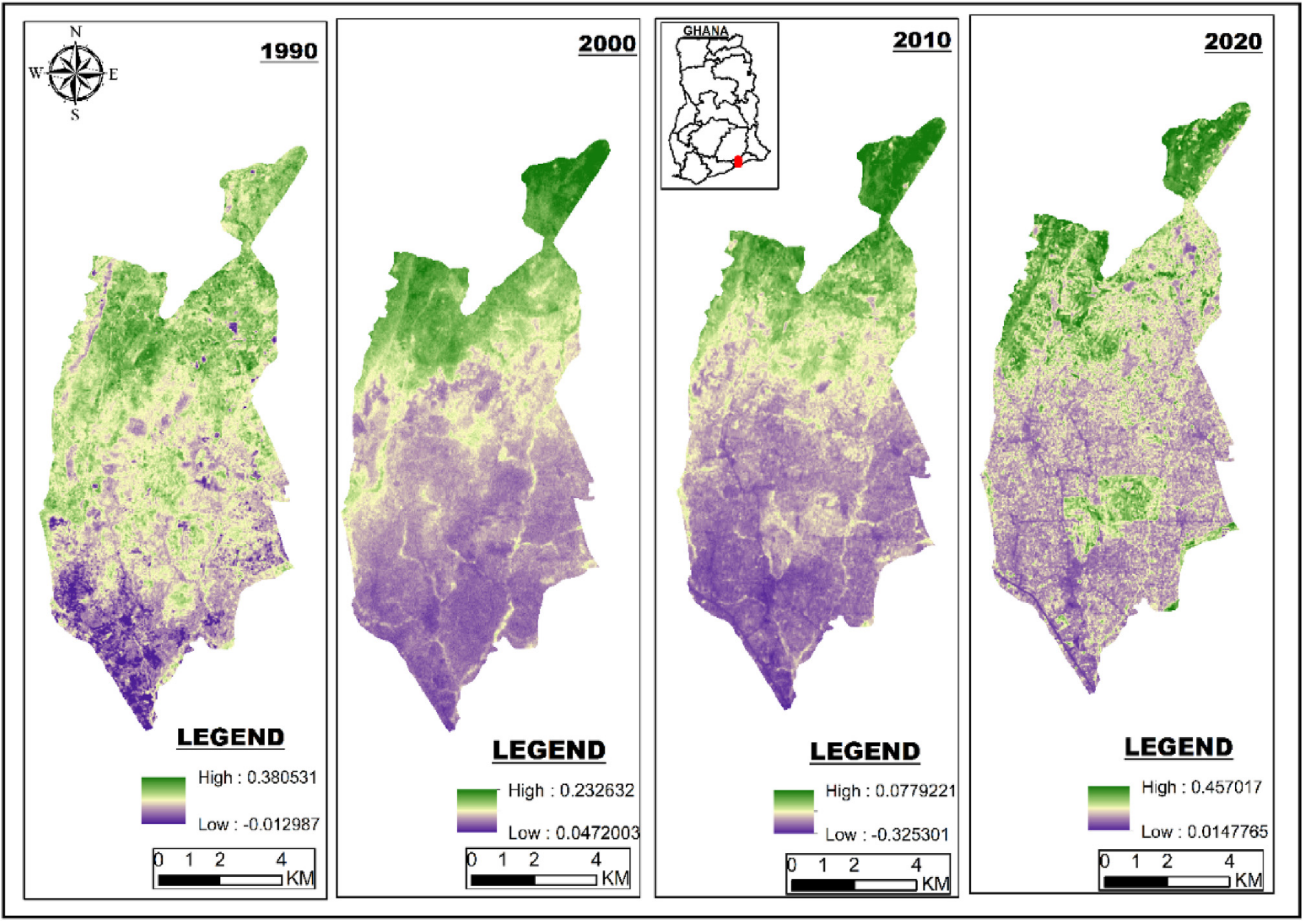
Normalized difference of vegetation Index.

The NDVI was examined to see the movement of vegetation in terms of how they have been removed, renewed or replanted over the years (Al-doski et al., 2013). In the Ga East Municipality, areas with healthy vegetation (closed, open thicket and dense herbaceous) are green colour (Figure 4). These healthy vegetated areas exhibit stronger-near-infrared reflectance, exemplifying high leaf biomass, canopy closure and higly chlorophyl vegetation (Lillesand et al., 2004) thus most of the visible light produce NDVI values ranging between 0.07 and 0.45. Contrarywise, areas with little of no vegetation such as grass, built-up surfaces, bare soil and rock were recorded in purple (Figure 4). These features reflect visible band more than near-infrared band. The NDVI or vegetative greenness was maximum in the extreme Northwest and Northeast of the Municipality ranging from 0.0779 to 0.4570. Whereas the South, Southwest and Eastern part of the Municipality are highly concretized hence having the least NDVI values ranging from −0.012 to 0.014 (with the exception of few patches being green in the central of the area). Built-up areas like St Johns, Dome, Kwabenya, Haatso Atomic in the South, southwest and Eastern part of the municipality have largely lower NDVI values such as 0.0472 and 0.00147. On the other hand, prominent patches green patches of NDVI of 0.232, 0.380 and 0.457 correspond to places like Sesemi, Abokobi, Boi and Adjankote hills all the northwest and northeast. This finding is similar to Grover and Singh (2015) who analyzed Urban Heat Island in relation to Normalized Difference Vegetation Index (NDVI) in Delhi and Mumbai.

### 4.2 Relationship between the LST, NDVI and LULC

The relationship between NDVI, LST and LULC were additionally appreciated by correlating their mean values for 1990, 2000, 2010 and 2020 with the corresponding percentage proportion of vegetation and non-vegetation. The results revealed a positive correlation between NDVI and LST (r = 0.8781, P < 0.133) where an increase in NDVI (as the area becomes more urbanized) leads to increase in LST. A negative correlation between the mean LST and the vegetated areas for all three decadal years (r = −0.67076, P < 0.003) was also presented. Thus, as the area covered by vegetation decreases, the mean LST increases. Again, there was a revelation of positive correlation between the mean LST and built—up areas (r = 0.716, P < 0.005), thus as the proportion of area covered by built increases, LST also increase. This finding is similar to Obiefuna et al. (2021), Igun and Williams (2018), Grover and Singh (2015), who found out that as cities like Lagos, Delhi and Mumbai become urbanized (increased built -up areas) LST rises.

### 4.3 Temperature and rainfall patterns and trend

As shown in previous studies in Ghana (e.g., Frimpong et al., 2017; Nunfam et al., 2021), the annual mean maximum and minimum temperature data over the last three decades indicates a steady increased warming trend and heat exposure (Figures 3c, d). Even though the annual mean temperature demonstrated a rising trend, they were highly erratic with some years of extremely high mean temperatures such as 1998, 2006, 2018 and 2020 which recorded 32.5 °C, 32.0 °C, 32.2 °C and 32.2 °C respectively. Similarly, 1991, 1996 and 2014 recorded the lowest annual mean maximum temperature of 31.0 °C. On the other hand, 2010 recorded the highest minimum annual mean with 24.5 °C and while 1992 and 1994 recorded the lowest annual minimum temperatures mean 24.4 °C each. Throughout these decades, the average maximum temperature increased by 1.5 °C. Nonetheless the greatest change in the average maximum temperature was recorded between 1990 to 2000 (1.5 °C). Comparably, the average minimum temperature increased by 1.1 °C within the same period with highest change of 0.9 °C occurring between 2000 and 2010. For the past three decades, the annual rainfall pattern varied considerably (Figure 3f; Table 2). Years of very high rains were 1991, and 2008 recording 1126.7 mm and 1161.7 mm respectively while 474 mm and 389.3 mm representing 1998 and 2013 respectively characterized years of very low rains. The annual average for these years has been 812.8 mm which in evaluation is slightly higher than the national annual average rainfall of 800 mm (World Bank Data, 2017). Some vivid distinctions marked by variable rainfall pattern (Table 2) were also observed. In the years 1991, 1996, 1999 and 2015 more rains were experienced during the dry months than minor seasons. In all, the mean dry season rainfall in January to March within the three decade were seen to be very erratic and highly variable. These variability has effects on heat stress on urban dwellers (Frimpong, K Oosthuizen and Van Etten, 2014; Frimpong, K Van Etten EJ, Oosthuzien and Fannam Nunfam, 2017).

### 4.4 Linking urban sprawl to climate change

Built up areas, Grass/shrubs and all vegetations (closed, opened thicket and dense herbaceous combined) were regressed against temperature and rainfall to establish some level of relationship. It came to light that there was strong statistical relationship between temperature and built up areas (r^2^ = 0.901, significant at the p < 0.05), grass/shrub (r^2^ = 0.911, significant at the p < 0.04) and all vegetations (r^2^ 0.923, significant at the p < 0.03). Thus as built up and grass/shrubs increases, temperature increases but as vegetation decreased temperature increases. Rainfall also showed strong statistical trend with built up (r^2^ = 0.940, significant at the p < 0.03), grass/shrub (r^2^ = 0.904, significant at the p < 0.04) and all vegetation (r^2^ = 0.954, significant at the p < 0.02). Whenever built up and grass/shrub increases annual average rainfall increase but vegetation decrease leads to rainfall increase. This corroborates Singh et al. (2020) who found a positive relationship between rainfall extremes and urbanization within a set of concentric ring buffers around rain gauge stations in the States, and Adeyemi et al. (2015) whose findings show that urban sprawl in a form of impervious surface have greater surface temperature than areas covered by vegetation.

### 4.5 Effects of urban sprawl on microclimate

**Plate 1:**
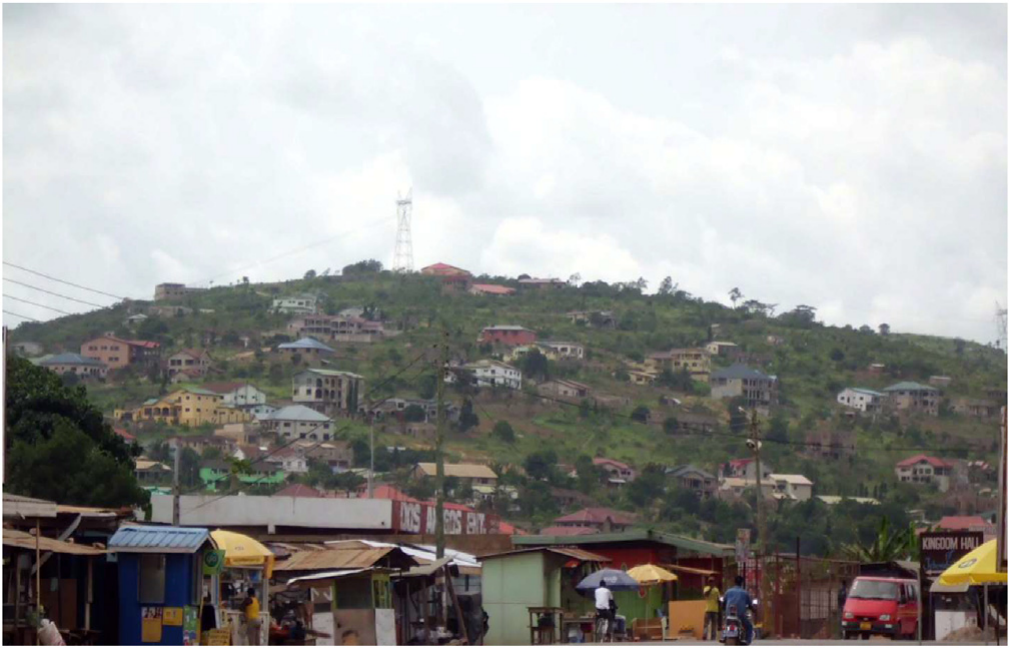
Built-up gradually taken over vegetation.

**Plate 2:**
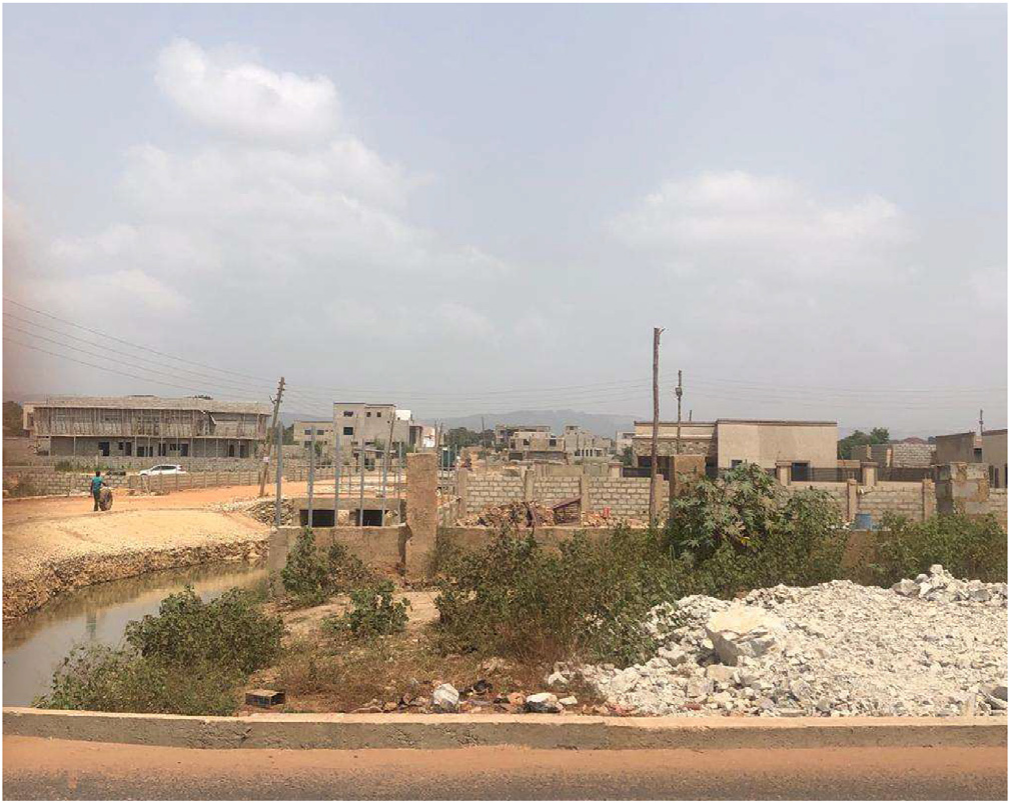
Effect of sprawling.

**Plate 3:**
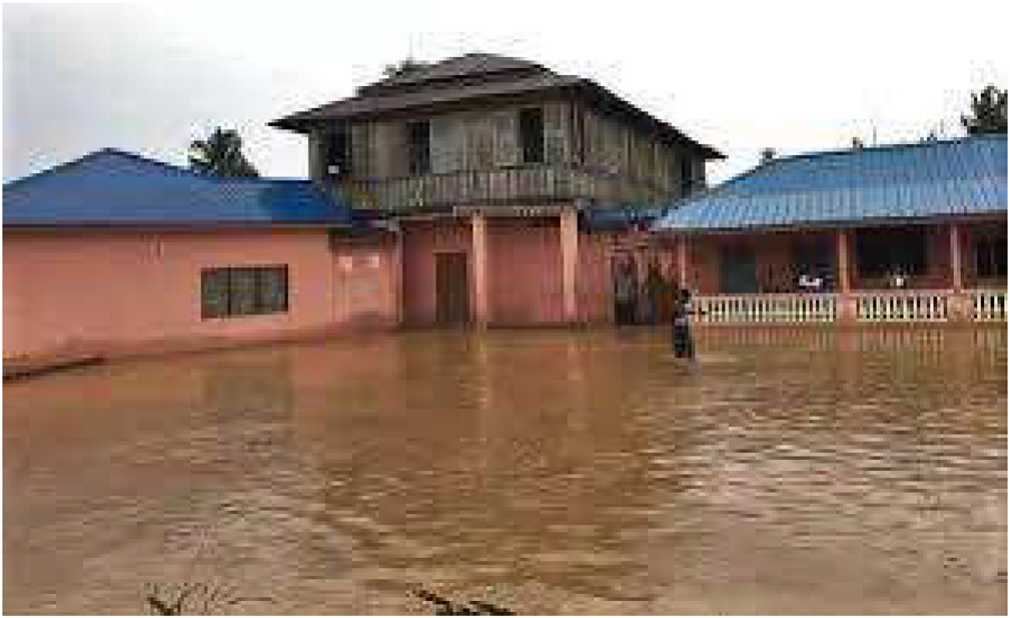
The study area experiencing floods.

**Plate 4:**
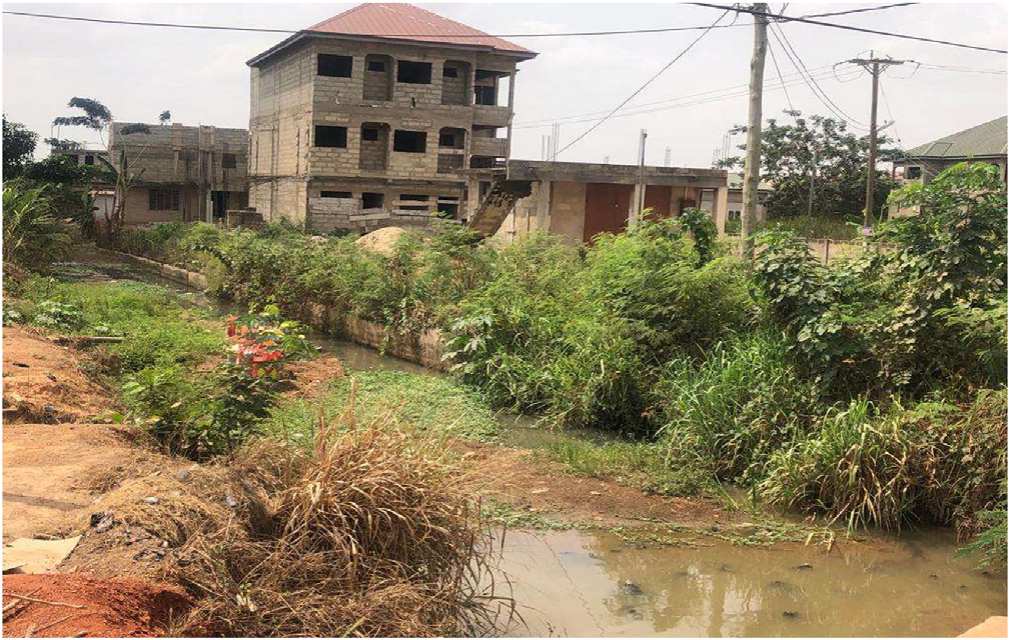
Building on a water course.

Climatic elements such as rainfall and temperature provide enormous support to human societies (Mensah, 2020), however these elements continue to be under serious threat through anthropogenic activities such as urban sprawl (Mattah et al., 2018; Li et al., 2017). The more urban population increases, the more space is needed to house these numbers. The overall effect is the conversion of vegetation in the form of green spaces and agricultural lands to built—up areas (Abass et al.*,* 2018; Emadodin et al., 2016). The conversion of land by sprawl has wielded impacts either directly or indirectly on climate change at the global state (Feng and Gauthier, 2021; Deng et al., 2013). The land cover matrix indicated that over the last 30 years, built up areas and grass/shrubs increased throughout (1.5 km^2^/years and 0.27 km^2^/years) respectively while the other land covers: closed thicket, open thicket and dense herbaceous reduced (- 0.17 km^2^/years, - 0.81 km^2^/years and—0.79 km^2^/years) throughout the same period (Figure 3 and Table 1). Temperature and rainfall from GMet have demonstrated rising trends over the last 30 years with mean maximum temperature increase by 1.5 °C and mean minimum temperature also increase by 1.1 °C. The annual average rainfall amounts have also increased by 8.8 mm over the same period. The reduction in vegetation at the expense of built up in the form of impervious surfaces could lead to increase land surface albedo which at the long run affect the microclimate (Boone et al., 2016; Sterling et al., 2013). There was high level of significance between urban sprawl and climate change in a form of land use and land cover changes. Thus, increased built up and grass/shrub led to increased temperature by 90% and 91% respectively, whereas a decreased vegetations led to an increased temperature by 92%. In terms of rainfall, urban built up and grass/shrub expansion caused annual average rainfall to also increase by 94% and 90% respectively. However, reduction in all vegetations led to an increase in the amount of annual average rainfall by 95%. An important thing to note is that often times reduction in vegetation and increased built-up have the tendency to reduce precipitation (Adler and Coauthors, 2017; Emadodin et al., 2016). However, this paper found that reduction in all vegetations as well as increased built-up (impervious surfaces) and grass/shrub rather leads to increase in rainfall. The reasons could be that urban sprawl has greater influence on temperature rather than rainfall. Again, in tropical Africa, rainfall is highly erratic and by its geographical position, rainfall in the Ga East Municipality is influenced more by other factors such as El Niño-Southern Oscillation (ENSO)—a natural phenomenon linked with sea surface temperature in the Tropical Pacific (Collins et al., 2010), the Atlantic sea surface temperature (SSTs) (Nutsukpo et al., 2012; Adler et al., 2000; Opoku-Ankomah and Cordery, 1994), the African Easterly Jet and the Tropical Easterly Jet (Price et al., 2007; Nicholson, 2008). Increased temperature and rainfall (r^2^ = 0.997, significant at p < 0.002) coupled with increased built-up areas (impervious surfaces) and grass/shrub could lead to floods with disastrous effects, breeding of some vectors like mosquitoes as well as extinction of some flora and fauna. While conversion of vegetation to urban built up could reduce evapotranspiration thereby changing the atmospheric balance. These results agree with other findings from Spracklen et al. (2018); Alkama and Cescatti (2016); Wang et al. (2017) that urban sprawl has the propensity to change the biogeochemical and biophysical effect that impact climate at all levels (see Plates 1, 2, 3, and 4).

### 4.6 Policy implication, recommendation and conclusion

The main aim of this paper was to assess the impact of urban sprawl on microclimate in the Ga East Municipality of Ghana for three decadal years using remote sensing and climate data. It was realized that the Ga East Municipality has undergone rapid urban sprawl especially built areas and grass/shrubs. The results indicate that between 1990–2020, built up areas and grass/shrub have increased by 45.4 km^2^ and 8.0 km^2^ respectively causing substantial reduction in all vegetations (closed thicket—5.21 km^2^ opened thicket—24.27 km^2^ and dense herbaceous—23.92 km^2^). Temperature and rainfall on the other hand also showed a steady increase in that while mean minimum temperature and mean maximum temperatures increased by 1.1 °C and 1.5 °C respectively, average rainfall amounts also increased by 8.8 mm over these decades. This reveals a momentous association between urban sprawl and climate change. The monolithic extension of built-up areas and grass/shrub in a form of impervious surfaces and the reduction in all vegetations led to increase surface albedo leading to increase in the average minimum and maximum temperatures as well as rainfall in the area within the period. These changes in climate brought about by urban sprawl will affect crop production, increase cataclysmic floods as well as growth of some harmful insects. The study requests the amalgamation of urban growth and climate change into spatial planning through an all-embracing approach. This will enable ease identification and utilization of the innumerable land use and land cover schemes leading to the overall progress of the local governance. There is also the need for awareness creation and directives on disorderly urban growth in the sense that inadvertent, haphazard and uncoordinated urbanization could lead disastrous changes in the environmental aspects like rainfall and temperature. Urban sprawl and climate change are broad phenomena which needs a poly-neutral consultation. Therefore, study calls for the inclusion and reinforcement of the various players to employ poly-neutral discussions together with bottom up approaches to ensure active dialogue regarding prioritization and implementation of plans.

## Declarations

### Author contribution statement

Frimpong Kwasi: Conceived and designed the experiments; Performed the experiments; Analyzed and interpreted the data; Contributed reagents, materials, analysis tools or data; Wrote the paper.

Eugene Atiemo Darko & Van Etten, E. J: Conceived and designed the experiments; Analyzed and interpreted the data; Contributed reagents, materials, analysis tools or data; Wrote the paper.

### Funding statement

This research did not receive any specific grant from funding agencies in the public, commercial, or not-for-profit sectors.

### Data availability statement

Data will be made available on request.

### Declaration of interest’s statement

The authors declare no conflict of interest.

### Additional information

No additional information is available for this paper.
